# Cancer: The Whole Story

**DOI:** 10.1371/journal.pbio.1001044

**Published:** 2011-04-12

**Authors:** Steven A. Frank

**Affiliations:** Department of Ecology and Evolutionary Biology, University of California Irvine, Irvine, California, United States of America

**Figure:**
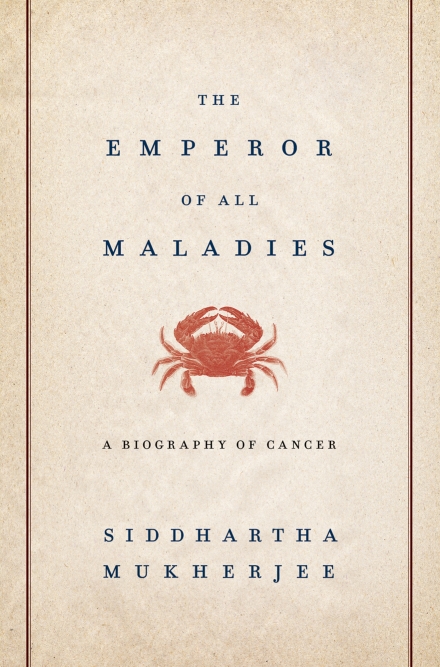
Mukherjee S (2010) The Emperor of All Maladies: A Biography of Cancer. New York: Scribner. 592 p. ISBN 978-1439107959 (hardcover). US$30.00.

For every patient and doctor, and for every scientist peering at a flask of deranged cells, this book connects the moment to the multiple voices that have played off each other since the first person squeezed a painful lump and wondered what to do.

Reading Siddhartha Mukherjee's *The Emperor of all Maladies: The Biography of Cancer*, the full accomplishment of this book slowly dawned on me. The story begins with a real patient with fulminant leukemia and inevitable terror, and a young doctor not sure of the course. The protocols of recent times are applied. But where did those treatments come from? The author, working in the Dana-Farber Cancer Institute and tracing back the history, comes to a hero of the past, Sidney Farber.

In the 1940s, what did leukemia seem like to Farber and to his patients? In fact, Farber, originally a pathologist, did not see patients. For the childhood leukemias that fascinated Farber, the children came to the hospital, were diagnosed, and over months died horrible deaths that devastated their families. There was no treatment. But Farber thought the right chemical combinations could be found to control and ultimately beat the disease. It was a heroic goal, but heroes often start as pariahs. And perhaps, in this case, the oncologists who shunned Farber had a point.

The theory of chemotherapy was simple. Poison the patient with chemicals that kill cells, and hope that cancer cells die faster than other cells. Hope was indeed a big part of the early studies. The chemical agents were potent poisons that worked very well, but their specificity for cancer cells as opposed to normal cells was not always so great. To knock the cancer back took a lot of poison, which was awful for the patient. Often, the only chance of knocking out the cancer required poisoning the patient right to, and too often past, the threshold of death. If the cancer was knocked out, the child had a brief reprieve. Soon enough the disease came roaring back, more aggressive and untreatable than before.

Oncologists wanted nothing to do with Farber, and did not want him “experimenting” on children in their hospital. The treatments were horrible, often more horrible than the disease itself. The supposed miracle cures created false hope, and then failed terribly. Farber was determined. He was more than determined. He *had* to find a way to treat and cure cancer. As with so many of the great characters in cancer's story, *no* was not an answer. It was an obstacle to be overcome, just like the disease itself.

Farber developed the chemicals, getting others to help. He came out of the lab, got some beds in the deepest, coldest, most isolated part of the hospital. At least, thought the other oncologists, don't let anyone see what he is doing. Farber recruited nurses and doctors, found patients whose families realized there was no other hope, guessed at some dosages, started injecting, and ran the ward. It was far from a clinical trial in the modern sense, but it was a real trial. Suffering and death were the norm, but that was already the baseline from which they started.

The author evokes the people, the failure, the eventual halting progress. Once Farber's voice has been introduced, the story moves off to develop other voices. But Farber continues to echo in the background. We may find ourselves in the modern Dana-Farber hospital walking by his old office, or hear Farber's spirit resonate with the personalities and the approaches of the great cancer surgeons who also tried to cure by first trying to destroy. How far toward death should the treatment go? How much horror in the often temporary cure justifies the journey through hell to get there? If it takes a great personal ego to smash through the obstacles of professional resistance to develop radical chemotherapy or radical surgery, can those giant egos learn and change as they are inevitably found to be partly right and partly wrong?

Patients, doctors, treatments. Heroes, dubious behavior, sometimes by the same people. This is already a rich story, beautifully told. The author has that very rare master's touch, evoking fully yet with the fewest of strokes. As readers, the experienced doctor, the bench scientist, and the patient will all move from sketch to realized story in different ways. There is detail and depth, but little to hinder.

With the patient-doctor-treatment counterpoint well established, the author adds new voices. Treatments through the 1960s progressed, clinical trial procedures were established, broad cooperative research programs emerged. But the success of treatments was confined to a few types of rare cancers. Overall, the total cancer burden changed little. Meanwhile, more was being learned about where the cancer burden came from. A lot came from cigarettes. We get the story of the epidemiological research, with new heroes.

Resistance always comes from somewhere. This time, it's the tobacco industry. You know the story in broad outline. The details resonate with what was being learned about the causes of cancer, with early detection through mammograms and the Pap smear, and with the complexities and controversies over the efficacy of screening. Competing interests arise and economics plays a role. There is increasing activism of the public in shaping research and health-related policies.

Until the 1970s, so little was understood about cancer and about how treatments worked, that it was all a black box. Presented with a disease, one poisoned or cut deeply and hoped the patient survived and the cancer died. By the 1970s, we learned to measure better and run proper trials, to cut a bit more or a bit less, to use different combinations of poisons. It was all empirical, in that little was really known about how different cancers differ and why individuals respond differently to the same treatments. And why, for many cancers, did death soon occur at nearly the same rate as before treatments existed?

Then we found some of the genes that mutated in cancers. We learned the biochemical actions of different potential treatments. Could we learn to match the specific changes in certain tumors to particular drugs designed to treat the specific malfunctions? Briefly, the answer is that we did in a few cases, we are still learning, and many people think great progress is ahead.

By this point in the story, you are well versed in the pace of history. At any time, always slow progress up to that point, new promise imagined ahead. But, as the author develops the story of recent research, you also feel the accelerating pace of change on top of that slow march through time. It was only 15 years ago that we first began to get any real genetic understanding, and those first clues were scattered and unclear. It was only a couple of years ago that we began to measure the actual genetic changes in tumors. And we know that genetic changes are only a part of the story. We have hints about the other factors, and just now can start to measure those factors such as epigenetic changes in DNA markings and histones, signaling changes between different cell types, and so on. The author brings us all the way to this point, keeping Farber and other early players alive through the narrative.

This book is about giving the full sense of time and pace and people. The narrative evokes detail rather than instructs. A reader expert in any area will see what is left out, what is made to sound simple when the reality is complex. But the whole story also has a reality, and there have been so very few authors who can tell us the whole story of major areas of medicine or science.

To tell the whole story, the author often focuses on individuals as heroes. The device works beautifully. Somehow, with a cast of Tolstoyian proportions, one can keep track of the individuals, and continue to hear their voices even as they come and go. I could not imagine another way to accomplish telling such a broad story, because we remember well-drawn characters long after we have forgotten about some particular technical achievement in a field far from our own. Yet, from the perspective of understanding the history of each era in a deeper and more nuanced way, it is probably good to keep a certain skepticism in mind.

In the subjects that I know well in cancer research, I think of the false tendency to exaggerate the role of a few individuals in ways that distort both the actual contributions of individuals and the actual way in which scientific understanding was achieved. The Nobel Prize winner Christian de Duve, when asked how he wanted to be remembered, answered:

I have no such ambition. In the history of science, my contributions are minor and would have been made by someone else had I not stumbled on them first. They already appear in textbooks without mention of my name. I am no Galileo, Newton, Darwin, Einstein or Watson and Crick. But I have had fun and have been rewarded beyond my deserts. So be it [1].

Nonetheless, a narrative following from one great person to the next is often a good way to tell the whole story:

More attention to the History of Science is needed, as much by scientists as by historians, and especially by biologists, and this should mean a deliberate attempt to understand the thoughts of the great masters of the past, to see in what circumstances or intellectual *milieu* their ideas were formed, where they took the wrong turning or stopped short on the right track. A sense of the *continuity* and the progressive and cumulative character of an advancing science is the best prophylactic I can suggest against the manic-depressive alternations of the cult of *vogue* and *boost*, which threatens to smother the scientific efforts, gigantic as they are, of at least one great nation [2].

Modern science naturally focuses itself almost entirely in the present and near future. But good treatment, research, and policy require a sense of the historical continuity and the progressive and cumulative character of advancing science—the whole story. To learn the whole story of cancer, read Siddhartha Mukherjee's masterful book.

**About the Author**

Steven A. Frank is Professor of Evolutionary Biology at the University of California, Irvine. He develops mathematical and computational models to study problems in evolutionary genetics, infectious disease, and cancer. Professor Frank has published three books: *Foundations of Social Evolution* (1998), *Immunology and Evolution of Infectious Disease* (2002), and *Dynamics of Cancer: Incidence, Inheritance, and Evolution* (2007). Further information about Professor Frank's research can be found on his Web site at http://stevefrank.org.
